# Metabolic Profiling Reveals Changes in Serum Predictive of Venous Ulcer Healing

**DOI:** 10.1097/SLA.0000000000004933

**Published:** 2023-01-10

**Authors:** Richmond T. Bergner, Sarah Onida, Rahul Velineni, Konstantina Spagou, Manjit S. Gohel, Marielle Bouschbacher, Serge Bohbot, Joseph Shalhoub, Elaine Holmes, Alun H. Davies

**Affiliations:** * Section of Systems Medicine, Department of Surgery and Cancer, Imperial College London, London, UK. Section of Vascular Surgery, Department of Surgery and Cancer, Imperial College London, London, UK;; † Academic Section of Vascular Surgery, Department of Surgery, Cancer, Imperial College London, London, UK;; ‡ Research, Development, Laboratories URGO, Chenôve, France; and; § Health Futures Institute, Murdoch University, Discovery Way, Perth, W.A., Australia

**Keywords:** mass spectrometry, metabolic profiling, metabolomics, NMR spectroscopy, venous leg ulceration, VLU, wound healing

## Abstract

The aim of this study was to identify potential biomarkers predictive of healing or failure to heal in a population with venous leg ulceration.

**Summary Background Data:**

Venous leg ulceration presents important physical, psychological, social and financial burdens. Compression therapy is the main treatment, but it can be painful and time-consuming, with significant recurrence rates. The identification of a reliable biochemical signature with the ability to identify nonhealing ulcers has important translational applications for disease prognostication, personalized health care and the development of novel therapies.

**Methods:**

Twenty-eight patients were assessed at baseline and at 20 weeks. Untargeted metabolic profiling was performed on urine, serum, and ulcer fluid, using mass spectrometry and nuclear magnetic resonance spectroscopy.

**Results:**

A differential metabolic phenotype was identified in healing (n = 15) compared to nonhealing (n = 13) venous leg ulcer patients. Analysis of the assigned metabolites found ceramide and carnitine metabolism to be relevant pathways. In this pilot study, only serum biofluids could differentiate between healing and nonhealing patients. The ratio of carnitine to ceramide was able to differentiate between healing phenotypes with 100% sensitivity, 79% specificity, and 91% accuracy.

**Conclusions:**

This study reports a metabolic signature predictive of healing in venous leg ulceration and presents potential translational applications for disease prognostication and development of targeted therapies.

Venous leg ulceration is a major public health concern, affecting up to 4% of those aged >65 years^1^ and costing up to 2% of the annual healthcare budget of Western societies.^2^ Patients experience chronic pain, embarrassment, and social isolation^3^; many are managed with long-term compression bandaging in the community,^4^ with poor healing rates and important recurrence rates. Even in the context of “best practice,” with patients undergoing intervention to abolish superficial venous reflux,^5^ ulcer healing is challenging to achieve and recurrence rates can be up to 20% within 1 year^5,6^; the mechanism for this is unclear.^7^ Venous leg ulceration is predicted to increase in prevalence with the ageing population^8^; it is therefore paramount to develop an understanding of factors that may influence ulcer healing and recurrence.

From a basic science perspective, a number of research efforts have explored the molecular biology of venous ulcer development and healing via omic approaches including genomics, transcriptomics, and proteomics; these have highlighted biological pathways of relevance but the results are yet to be translated into the clinical arena.^7^ Metabolic phenotyping, the study of the end products of cellular metabolism, is relatively novel technology that enables qualitative and quantitative assessment of metabolites associated with a disease process, helping identify novel diagnostic and prognostic biomarkers. Platforms such as nuclear magnetic resonance spectroscopy (NMR) and mass spectrometry (MS) permit a global, in-depth analysis of the metabolome by providing complementary information on aqueous and lipid metabolites.^9–11^

This information is also complementary to data obtained via other omic approaches and can help develop applications to improve patient care. Metabolic phenotyping has had limited applications to venous leg ulceration^12^; reported metabolites of relevance include L-arginine, nitric oxide, iron, and free radicals, which are increased in ulcer fluid samples,^12^ supporting proteomic data describing an inflammatory phenotype in these wounds once they are established.^13,14^ Despite this wealth of data, there are no studies exploring metabolites associated with, or predictive of a healing phenotype, which would have major clinical applications in terms of patient management.

The authors hypothesized that metabolic profiling of baseline biofluid samples would predict ulcer healing status. The aim of this pilot study was to identify metabolic features predictive of venous leg ulcer healing or failure to heal at 20 weeks via NMR and MS analysis. The identification of predictive biomarkers may have important translational applications for disease prognostication and the development of novel therapies.

## Methods

Participants were recruited prospectively at four sites: Imperial College Healthcare NHS Trust, Central London Community Hospitals NHS Trust, Cambridge University Hospitals NHS Foundation Trust, and West Suffolk NHS Foundation Trust.

### Endpoints

The primary endpoint was healing status at 20 weeks. Secondary endpoints included change in ulcer size, difference in ulcer age and in phenotype.

### Recruitment

This was a pilot study to assess the ability to identify bio-markers predictive of healing status via metabolic profiling. The sample size was based on previously reported studies,^12^ which recruited approximately 20 patients. For the purposes of this study, it was estimated that at least 30 patients would be required, including loss to follow-up.

Patient selection was based upon the following inclusion criteria: age >18 years, presence of chronic venous ulceration (defined as an ulcer present for ≥4 weeks), ankle brachial pressure index >0.85, and venous duplex ultrasound evidence of venous insufficiency.^15^ Any patients with acute infection of the affected limb in the preceding 4 weeks, a history of connective tissue disease, or immunosuppressive medications (corticosteroids, chemotherapy, radiotherapy, recombinant immunological medications) were excluded from the study.

**Figure 1:**
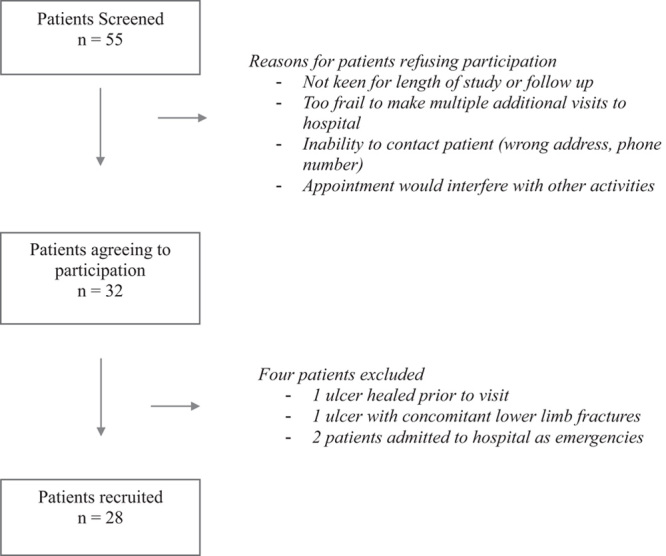
Screening and recruitment workflow.

Fifty-five screened patients with venous leg ulceration and documented venous reflux (superficial or deep) on duplex ultrasound were identified using local databases and clinic lists; these were approached and provided with information on the study, with full informed consent taken from those willing to participate. Thirty-two patients consented, enabling the authors to meet the recruitment target. Patients were excluded as per the screening and recruitment workflow (Fig. 1); in total, 28 participants were included in the analysis. All participants underwent a comprehensive history and clinical examination. Ulcer size and surface area were recorded and baseline blood, urine, and ulcer fluid samples were collected for all participants. At week 20, ulcer size and healing status were recorded;

in total, 15 patients had healed and 13 had not. Ulcer healing status was chosen as a dichotomous outcome for assessment of metabolite differences.

### Sample Collection

Samples were collected according to departmental standard operating procedures. Participants were not fasted before sample collection.

#### Urine

Patients were asked to provide a mid-stream urine sample into a 50 mL Falcon tube (Beckton Dickson and Company, Franklin Lakes, NJ). Three 1 mL aliquots of urine were collected into 1.5 mL Eppendorf tubes, immediately placed on dry ice and transferred to a − 80^o^C freezer.

#### Serum

Antecubital fossa venepuncture was performed using a 21G needle and 10 mL syringe. Blood was transferred into two 13 × 100 mm (5 mL) plastic serum tubes (Beckton Dickson and Company, Franklin Lakes, NJ), allowed to stand for 30 minutes at room temperature, centrifuged at 2500 relative centrifugal force for 10 minutes and a minimum of 0.4 mL of serum fraction was transferred into three 1.5 mL Eppendorf tubes; these were immediately placed on dry ice and transferred to a −80^o^C freezer.

#### Ulcer Fluid

Wounds were irrigated with 0.5 mL of sterile water. Based on ulcer size, up to 3 sterile 1 cm diameter filter discs (Sigma-Aldrich, St. Louis, MO) were placed in the wound bed and left for up to 90 minutes while covered with an occlusive dressing. Filter discs were collected upon dressing removal and placed into individual Eppendorf tubes. Samples were immediately placed in dry ice and transferred to long-term storage at −80^o^C. After sampling, the affected limbs were dressed and placed in compression as per standard care by a trained specialist nurse.

### Experimental Analysis

#### Sample Preparation and Ultra-Performance Liquid Chromatography-Mass Spectrometry Analysis

Serum and urine samples were analyzed by reversed phase (RP) and hydrophilic interaction liquid chromatography (HILIC) ultra-performance liquid chromatography-mass spectrometry (UPLC-MS) profiling methods. For ulcer fluid, organic and aqueous metabolites were extracted using a modification of a previously described protocol.^10^ Lipid profiling of serum and ulcer fluid organic metabolite extracts was performed using RP-UPLC-MS as previously described.^16^ Serum and urine HILIC-UPLC-MS metabolic profiling was conducted using a modification of the protocol as described in the supplemental material (see Text, Supplemental Digital Content 1, Expanded methods, http://links.lww.com/SLA/D134). The separated samples were analyzed with electrospray ionization (ESI) + and ESI − modes (see Text, Supplemental Digital Content 1, Expanded methods, http://links.lww.com/SLA/D134).

#### Sample Preparation and NMR Analysis

For NMR analysis of serum and ulcer fluid, aqueous extract buffer solution of 0.075 M Na_2_HPO_4_ × 7H_2_O, 4% sodium azide (NaN_3_)inH_2_O, 20% deuterium oxide (D_2_O), and 0.08% 3-(tri-methyl-silyl) propionic acid-d4 (TSP) was added to serum and ulcer fluid aqueous extract samples. For NMR urine analysis, 0.075 M Na_2_HPO_4_ × 7H_2_O, 17% deuterium oxide (D_2_O) containing 0.1% TSP was added to the urine samples. ^1^H-NMR data acquisition was performed as previously described^17^ (see Text, Supplemental Digital Content 1, Expanded methods, http://links.lww.com/SLA/D134).

#### Statistics

Selected characteristics of the study populations were described and compared according to healing status. Differences between healing groups were tested using the Kruskal-Wallis 1-way analysis of variance (ANOVA).

The raw MS data were collected in centroid mode and converted to netCDF format using the DataBridge tool implemented in MassLynx software (Waters Corporation, Milford, USA). The data were pre-processed using the XCMS^18^ package in R programming software; an output table was obtained comprising pairs of mass/charge ratio (m/z), retention tim, and intensity values of the detected metabolite features in each sample. Integrals of metabolic features with coefficient of variation >30% in the quality control (QC) samples were removed. The resulting data underwent total area normalization. For the NMR dataset, spectral data were imported into MATLAB R2014a (MathWorks, USA) for preprocessing. The regions corresponding to water, urea, and TSP were excised and the remaining spectra were calibrated and probabilistic quotient normalized using in-house developed scripts. Each dataset was analyzed via SIMCA 14 (Umetrics, Sweden) to highlight the difference between the experimental groups and to identify key discriminatory metabolic features. Principal component analysis (PCA) was applied to assess instrument stability, clustering of samples, and separation of experimental groups, as well as to detect analytical or biological outliers. Orthogonal partial least squares discriminant analysis (OPLS-DA) with significance testing by ANOVA of the cross-validated residuals (CV-ANOVA) was performed, using the orthogonal filter to remove extraneous systematic variance in the data.^19^ Univariate statistical analysis was applied to each discriminatory metabolite identified from the multivariate modeling followed by a 2-tailed Student *t* test with a Benjamini-Hochberg correction for multiple testing (adjusted *P* value). An adjusted *P* value of <0.05 was defined as significant.

Metabolite identification by MS was conducted by matching accurate m/z measurements of detected chromatographic peaks and molecular fragments using the literature,^20^ Kyoto Encyclopedia of Genes and Genomes,^21^ LIPID MAPS,^22^ and METLIN.^23^ Tandem MS fragmentation patterns were performed to obtain further structural elucidation. NMR-derived metabolites were identified using in-house databases and the published literature.^24^

#### Study Approval

The study was conducted in accordance with the Declaration of Helsinki principles. Ethical approval was obtained from the East of England Research Ethics Committee (REC 13/EE/0137). Written informed consent was obtained from all participants before inclusion in the study.

## Results

### Patient Demographics

Demographic and clinical data are presented in Table 1. At baseline assessment, all participants were C6 class as defined by the Clinical, Etiology, Anatomy, Pathophysiology (CEAP) classification. In 17 patients (60.7%), the underlying hemodynamic abnormality was primary superficial venous reflux; in 1 (3.6%) primary deep reflux; in 10 (35.7%) secondary deep reflux (due to a historical deep vein thrombosis). None of the patients had an obstructive picture on their duplex ultrasound assessment and there was no difference in the distribution of hemodynamic disease patterns between the healer and nonhealer groups.

**Table 1:** Patient Demographics and Ulcer Features. There Were No Statistically Significant Differences in Age Distribution or Sex Ratio Between Healed and Nonhealed Groups Analyzed. There Were Significant Differences in Ulcer Area, Perimeter, and Age Between Healed and Nonhealed Groups

	Healed Ulcer (N = 15)	Nonhealed Ulcer (N = 13)	*P*
Age, y, (SD)	73 (12)	71 (16)	0.7191
Male, n (%)	9 (60)	9 (69)	0.5024
Ulcer area, cm^3^, (SD)	27 (38)	65 (50)	**0.0333**
Ulcer perimeter, cm, (SD)	13.6 (15.7)	27.86 (16.48)	**0.0281**
Ulcer age, mo, (SD)	27 (39)	65 (50)	**0.0333**

n indicates number; SD, standard deviation.

Participants were managed with compression therapy, with none undergoing additional venous interventions during the study period. Although patient age and sex were not statistically significantly different between healers and nonhealers, ulcer size and ulcer age were.

### Metabolic Profiling Analysis

The metabolic profiling analysis of serum, urine and ulcer fluid samples generated 13 datasets. Five experiments were performed for urine and serum analysis: NMR, RP UPLC-MS in ESI+ and ESI−, HILIC-UPLC-MS in ESI+ and ESI−. Three experiments were performed for each ulcer fluid sample: NMR and reversed-phase UPLC-MS in ESI+ and ESI−.

### Reversed Phase UPLC-MS Analysis of Serum, Urine, and Ulcer Fluid of the Venous Leg Ulcer Patients

Serum, urine, and ulcer fluid samples were analyzed using reversed phase UPLC-MS identifying the following metabolic features: 8296 in serum positive ionization mode (ESI+), and 2929 in serum negative mode (ESI−); 5263 in urine ESI+ and 7026 features in ESI−;16,457inulcer fluid ESI+ and 7204 in ESI−. Contaminant peaks in either QCs or blank controls were excluded and the retained metabolic features were used for the final analysis. The robustness of the serum, urine, and ulcer fluid MS experiments was confirmed as QC samples showed clustering in the PCA models (see Figure, Supplemental Digital Content 2, Figures 1–6. PCA models, http://links.lww.com/SLA/D134).

PCA models for the RP-UPLC-MS profiles showed no obvious separation between the healed and nonhealed groups in serum, urine, and ulcer fluid in either ionization mode (see Figure, Supplemental Digital Content 2, Figures 1–6, PCA models, http://links.lww.com/SLA/D134); conversely, OPLS-DA models highlighted separation according to healing phenotype.

**Figure 2:**
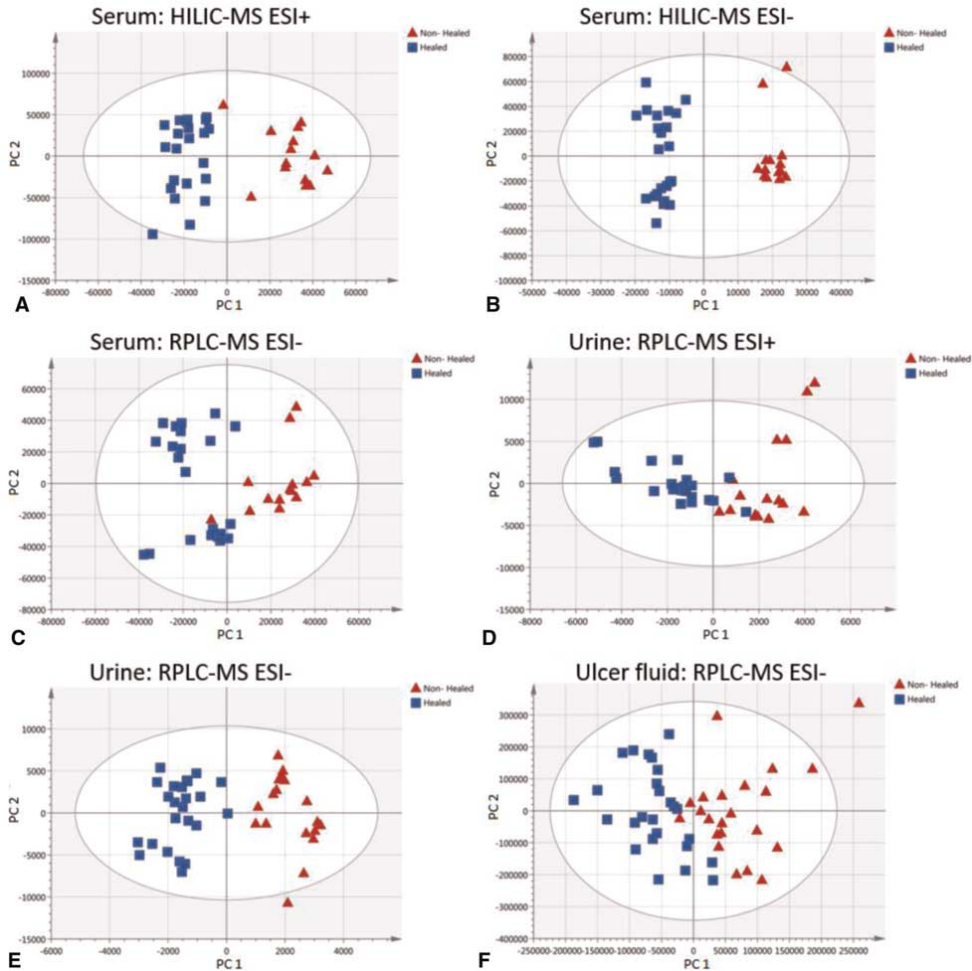
Multivariate OPLS-DA cross-validated scores for metabolic profiling of serum, urine and ulcer fluid samples of non-healed (red) and Healed (blue) vein leg ulcer patients. (A) positive mode (ESI+) HILIC-MS and (B) negative mode (ESI−) HILIC-MS profiling analysis of serum; (C) negative mode (ESI−) of lipid RPLC-MS profiling analysis of serum; (D) positive mode (ESI+) RPLC-MS and (E) negative mode (ESI−) of RPLC-MS profiling analysis of urine; (F) negative mode (ESI−) of lipid RPLC-MS profiling analysis of ulcer fluid. The *R* ^2^Y and *Q* ^2^Y values for OPLS-DA scores plots are shown in Table 3.

The significance and robustness of the corresponding OPLS-DA models for serum (ESI−, *P* = 0.0005 for R^2^Y = 90% and Q^2^Y = 57%), urine (ESI+, *P* = 0.046 for R^2^Y = 64% and Q^2^Y = 25% and ESI−, *P* = 0.024 for R^2^Y = 87% and Q^2^Y = 31%) and ulcer fluid (ESI−, *P* = 0.047 for R^2^Y = 58% and Q^2^Y = 20%) were confirmed by CV-ANOVA testing (Table 2 and Fig. 2). All other RP-UPLC-MS profiles showed no significant difference between groups. The S-plot in SIMCA was used to visualize metabolic features contributing to differences in the significant OPLS-DA models. Once univariate statistical testing was conducted on the integrals of the metabolic features responsible for the class discrimination (ie, healed vs no-healed) for urine and ulcer fluid discriminatory metabolic features, none of these remained statistically significant following Benjamini-Hochberg multiple testing correction. Discriminatory features derived from serum data remained significant. Overall, the serum lipid profiling analysis revealed 8 unique discriminant features between groups of interest. These included 6 ceramides (Cer d18:1/24:0, d18:1/24:1, d18:1/23:0, d18:2/23:0, d18:1/22:0, and d18:2/22:0), 1 ceramide-1-phosphate (CerP d18:1/18:0), and 1 sphingomyelin (SM d18:1/23:0). Overall, 6 different ceramide and ceramide-1-phosphate (CerP d18:1/18:0) intensities were shown to be significantly reduced in the healed versus the nonhealed patients. SM d18:1/23:0 was found to be have significantly reduced intensities in the healed versus nonhealed group (Table 3).

**Table 2:** Summary of Model Characteristics From Significant OPLS-DA Multivariate Statistical Analysis of all Analyzed Data

Sample Type	Analysis	Components	*R* ^2^X	*R* ^2^Y	*Q* ^2^Y	CV ANOVA
Serum	HILIC-MS ESI+	1 + 2	47.20%	86.00%	43.90%	6.66E-03
	HILIC-MS ESI−	1 + 2	24.70%	96.60%	34.20%	3.76E-02
	RPLC-MS ESI−	1 + 1	35.50%	90.40%	57.10%	5.25E-04
Urine	RPLC-MS ESI +	1 + 1	24.20%	64.40%	24.90%	4.55E-02
	RPLC-MS ESI−	1 + 2	27.30%	87.40%	31.90%	2.78E-02
Ulcer fluid	RPLC-MS ESI−	1 + 1	30.30%	58.10%	19.70%	4.72E-02

*Q* ^2^ indicates predictive ability of the model; *R* ^2^, variation in the matrices explained by the model; RPLC-MS, reversed phase liquid chromatography-mass spectrometry.

**Table 3:** Discriminatory Metabolites Observed in Serum Via UPLC-MS. Significance Measured Via Student *t* Test With Benjamini-Hochberg Multiple Testing Correction

Metabolite	Fold Change (H/NH)	*P*	Adjusted *P*
Carnitine	1.840284485	8.76E-04	3.75E-02
Car (d18:1/24:0)	0.790378672	3.18E-04	2.80E-02
CerP (d18:1/18:0)	0.782541073	9.10E-04	4.17E-02
Cer (d18:2/23:0)	0.781021223	3.35E-04	2.80E-02
Cer (d18:1/22:0)	0.744297674	2.45E-04	2.68E-02
Cer (d18:1/24:1)	0.738650889	1.23E-03	4.88E-02
Cer (d18:1/23:0)	0.722764682	1.97E-04	2.68E-02
Cer (d18:2/22:0)	0.656515972	2.32E-04	2.68E-02
SM (d18:1/23:0)	0.654286489	9.28E-04	4.17E-02
PE (18:4/22:6)	0.349720924	9.68E-04	3.75E-02

Cer indicates ceramide; H, healers; NH, nonhealers.

### HILIC-UPLC-MS Analysis of Serum, Urine, and Ulcer Fluid of the Venous Leg Ulcer Patients

Serum and urine samples were examined using HILIC-UPLC-MS. Overall, 4302 metabolic features were identified in serum ESI+ and 3623 in ESI−; in urine 15,637 metabolic features in ESI+ and 5491 in ESI−. This resulted in a remainder of 3410 features for ESI+ and 2442 features for the ESI− on serum analysis. In urine analysis, ESI+ detected 12,394 features and ESI− 3701 features. These metabolic features were employed for the following analyses. For both the serum and urine profiles, PCA models revealed close clustering of QC samples (see Figure, Supplemental Digital Content 2, Figures 1–6, PCA models, http://links.lww.com/SLA/D134). This confirmed the validity and robustness of the MS experiments. Profiles acquired from both serum and urine samples did not reveal any inherent separation between healers and nonhealers in the first 2 PCA components in both ionization modes.

OPLS-DA models revealed differences in serum metabolic profile, with CV-ANOVA testing confirming model robustness (ESI+, *P* = 0.006 for R^2^Y = 86% and Q^2^Y = 44% and ESI-, *P* = 0.034 for R^2^Y = 97% and Q^2^Y = 34%). However, none of the profiles derived from the urine samples were significant (Table 2).

Further statistical testing was performed on the integrals of the metabolic features that differentiated between classes (ie, healed vs nonhealed) in the serum matrix. Only 2 unique metabolic features maintained statistical significance following Benjamini-Hochberg correction for multiple testing. These significant included L-carnitine and phosphatidylethanolamine (PE 18:4/22:6). In total, the integral intensity of carnitine was significantly elevated in the healed group compared to the nonhealed group. Furthermore, PE 18:4/22:6 intensity was significantly reduced in the healed patient serum samples compared to the nonhealed samples.

### ^1^H-NMR Metabolic Profiling of Serum, Urine, and Ulcer Fluid From Venous Leg Ulcer Patients

The metabolic profiles of serum, urine, and ulcer fluid were separately analyzed using 1-dimensional ^1^H-NMR. Acetaminophen and ethanol were removed from all spectra before analysis since these exogenous metabolites do not relate to biological responses. To identify metabolite patterns associated with healing status a multivariate approach was undertaken. PCA models confirmed experiment validity, revealing tight clustering of all QC samples; however, no separation was seen between healers and nonhealers for all biofluids. OPLS-DA models also failed to demonstrate a significant separation of healers versus nonhealers following CV-ANOVA testing. Moreover, the metabolite signatures did not show significant associations to patient age, sex, ulcer size, and ulcer age.

### Ratio of Carnitine to Ceramide as a Serum Biomarker Predictive of Ulcer Healing

**Figure 3:**
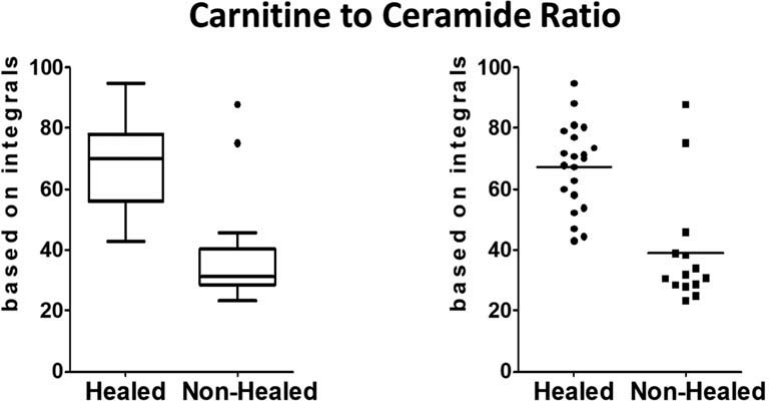
Carnitine to ceramide ratio as a predictive biomarker of ulcer healing. Significance measured via Student unpaired *t* test (*P* < 0.0001).

The detection of elevated serum carnitine and decreased ceramide levels associated with ulcer healing via UPLC-MS highlighted the theory that these metabolites may potentially have opposing roles in venous leg ulcer healing. Hence, the utility of a serum-based carnitine to ceramide ratio was investigated as a potential prognostic biomarker for venous leg ulcer healing. Patients with healed leg ulcers manifested a significantly elevated carnitine to ceramide (total) ratio (*P* < 0.0001) compared to non-healing patients (Fig. 3). The use of carnitine/ceramide ratio as a prognostic marker was able to distinguish the healed from nonhealed leg ulcer patients with 100% sensitivity, 79% specificity, and 91% accuracy.

## Discussion

The healing phenotype was significantly associated with metabolic differences in serum derived profiles, but not urine or ulcer fluid derived profiles. The serum-derived profiles of the RP and

HILIC-UPLC-MS, but not ^1^H NMR, showed potential of this experimental approach in the development of prognostic markers for ulcer healing.

The serum discriminatory profile was driven by amino acid derivatives and lipids. Serum _L_-carnitine was the single, most significantly altered metabolite detected by HILIC-UPLC-MS ESI−. _L_-carnitine was increased (1.84-fold) in the healed versus nonhealed group. Raised serum levels have been associated to malnutrition^25^ dilated cardiomyopathy,^26^ atherosclerosis^27^ and myocardial infarction.^28^_L_-carnitine is an important nutrient that contributes to energy and fatty acid metabolism; it facilitates the transport of activated fatty acids across the mitochondrial intermembrane into the mitochondrial matrix to induce β-oxidation and generate energy.^29^ Carnitine supplementation has been reported to enhance performance in athletes.^30^ In murine experimental models of skin flaps and wounds, carnitine supplementation has positive effects on healing rates,^31^ skin tensile strength, and angiogenesis.^32^ Isolated case reports have described its beneficial effects in leg ulcer patients^33^; however, no significant effects were noted in the only pilot randomized trial performed,^34^ which was described as low quality by a Cochrane review.^35^ Elevation of carnitine in the serum of healed patients in this study may potentially suggest increased carnitine levels at the ulcer site to support the generation of new cells and healing^36^; this may also explain the reduced ceramide, sphingomyelin, and phosphatidylethanolamine levels, which are cell membrane components.^37^ Alternatively, elevated _L_-carnitine levels may suggest an improved ability to respond to trauma and recover from injury; dysregulated plasma carnitine levels have been described in malnutrition and chronic disease.^38^

Elevated carnitine levels were not detected in the ulcer fluid of healers in this study; this may be due to lack of statistical power. The reason for not detecting elevated carnitine in the urine samples of healed patients may be due to the renal absorption of 98% to 99% of circulating carnitine filtered at the glomerulus.^39^ This is the first study reporting an association between carnitine levels and healing status in venous leg ulceration, with the aim of identifying a biomarker predictive of ulcer healing. The authors have previously described articles characterizing the metabolic phenotype of venous ulceration in tissue, reporting metabolites associated with bacterial, nucleotide, energy metabolism, and cellular destruction.^9^ Carnitines have been described in the context of venous thromboembolism; reduced acylcarnitine levels are associated with a prothrombotic status and increased risk of venous thromboembolism, due to their anticoagulant activity as inhibitors of factor Xa.^40^ Both L-carnitines and acylcarnitines are important in maintaining normal mitochondrial function.^41^ None of the participants in this study had a history of venous thromboembolic events; it is likely that the association between L-carnitine and healing status is related to energy metabolism.

Other significant differences in the metabolic profiles of the serum samples included ceramides (Cer d18:1/24:0, d18:1/24:1, d18:1/23:0, d18:2/23:0, d18:1/22:0, and d18:2/22:0), which were reduced in healers compared to non-healers. Ceramides are lipids composed of sphingosines attached to fatty acids. Recent literature has revealed them to be increased in almost all stress stimuli including inflammation, heat, ultra violet light, hypoxia, and oxidative stress.^42^ The de-novo synthesis of ceramide requires fatty acid transfers on a serine residue and a sphinganine. As elevated levels of activated fatty acids are channelled into β-oxidationviacarnitine, the activated fatty acid availability in the healed patient group may be reduced for ceramide synthesis. Furthermore, recent literature has shown that ceramides have a wide impact on cellular metabolism,^43^ preventing lipid and amino acid uptake by blocking specific transporters, thereby blocking cellular protein synthesis.^44^ Overall, this may explain how reduced ceramides levels in healers may facilitate nutrient uptake to support ulcer healing by permitting nutrient transporter function, contributing to protein synthesis required for wound healing. Another major mechanism by which ceramides alter cellular metabolism is via the inhibition of Akt,^45^ a serine/threonine kinase that activates signaling pathways involved in cell growth and subcellular distribution of nutrient transporters. Akt activates anabolic pathways while attenuating catabolic ones. Ceramides regulate Akt action by mediating dephosphorylation of Akt, rendering it inactive. In addition, ceramides prevent Akt translocation, rendering it nonfunctional.^46^ As such, reduced ceramide levels in the healed patient group may allow for Akt activation to drive ulcer healing. This is further supported by the literature, as activation of the Akt/mTOR signaling pathway contributes to wound healing.^47^

**Figure 4:**
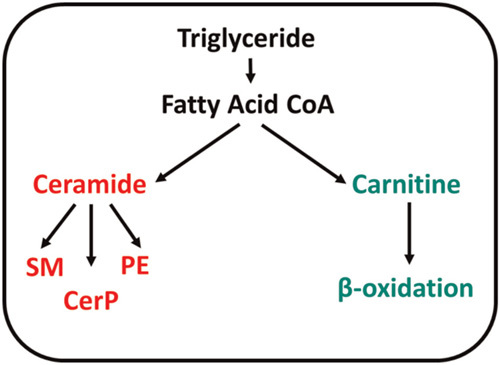
Predicted altered carnitine and ceramide metabolic pathways in serum samples obtained from the healed venous leg ulcer group. Green indicates metabolites increased in healed group compared to nonhealed group whereas red indicates decreased levels of metabolites.

The serum levels of CerP (d18:1/18:0), SM (d18:1/23:0), and PE (18:4/22:6) were reduced in healers compared to nonhealers. All three are downstream metabolites of ceramide, hence, their reduction may be explained by the decreased ceramides in the healed versus nonhealed group (Fig. 4).

In summary, this pilot study is the first to employ a global metabolic profiling approach to assess the prognostication of venous ulcer healing status using serum, urine, and ulcer fluid. Few authors have employed the aforementioned experimental platforms to investigate venous leg ulceration; NMR has previously been utilized to describe the phenotype of ulcer tissue and ulcer fluid.^9^

Robust models generated from ^1^H-NMR, RP, and HILIC-UPLC-MS platforms allowed for this exploratory examination. Although urine and ulcer fluid-derived profiles failed to detect significant metabolic differences, serum-derived profiles revealed significant differences between healed and non-healed patients. The lack of a defined urinary phenotype of healed versus nonhealed ulceration may be due to lack of statistical power, particularly in a biofluid whose composition, unlike serum, is not maintained under tight homeostatic control. The lack of an ulcer fluid phenotype may also be due to the relatively small sample size in this pilot study. Another possible explanation is that wound healing may be influenced by systemic, in addition to local, factors. In other words, different individuals may have a different likelihood of “healing” veesus “nonhealing” based on their inherent characteristics and how these interplay with their ulcer phenotype. It is also possible that wound fluid may not be the most appropriate medium to analyse the local wound microenvironment and that biopsy of the healing/non-healing ulcer edge may provide more in-depth information regarding the local biological processes. Future studies should consider the analysis of tissue biopsies from the ulcer edge, whether by homogenization or topographical metabolic profiling, to better characterize the pathways involved in healing in a larger sample size.

Serum carnitine has been implicated in cardiovascular disease.^48^ This study suggests that the increased serum carnitine to ceramide ratio, which highly correlated with the healed phenotype, may be a promising candidate biomarker. Validation and implementation of this prognostic biomarker for prediction of healing based on the results of this pilot study is required to enable improved stratification and clinical management of venous leg ulcer patients. Furthermore, future assessment of systemic biomarker levels beyond ulcer healing would help clarify their role in venous ulceration and in ulcer recurrence. Carnitine supplementation in murine models contributes to enhanced wound healing.^31^ Further prospective studies investigating the role of carnitine in wound biology would be of interest to ascertain whether carnitine supplementation at a local or at a systemic level may aid venous leg ulcer healing.

This pilot study provides important insights on metabolites and pathways that may have a prognostic role in identifying patients more or less likely to heal their venous leg ulcer. There are some limitations to this study. The recruited sample size is limited, which may have led to the lack of discriminating metabolites in ulcer fluid and upon ^1^H-NMR analysis. Future validation work will require a larger sample size to describe in greater detail the predictive ability of the identified metabolites. Finally, the analysis of the local ulcer microenvironment via ulcer fluid analysis did not yield significant results; this may be due to the sample size or to the choice of substrate. It is possible that tissue biopsies of the healing/nonhealing wound edge interface will provide more information than wound fluid; future work should consider including these samples in the analysis.

## Supplementary Material
