# Supplementary material for: Metabolic Profiling Reveals Changes in Serum Predictive of Venous Ulcer Healing
Source: Ann Surg. 2023 Jan 10;277(2):e467–74. doi: 10.1097/SLA.0000000000004933 (PMC9831039; doi:10.1097/SLA.0000000000004933)
Supplement: Supplementary file 1 [file sla-277-e467-s001.docx]

**SUPPLEMENTAL DIGITAL CONTENT**

**1. Expanded Methods**

**Ulcer Fluid Metabolic Profiling Analysis**

**Metabolite Extraction**

The extraction of polar and non-polar metabolites from the ulcer fluid and filter discs was performed simultaneously using a biphasic separation protocol (two-layer extraction strategy), by adding 700μL of solvent mixture of methyl tert- butyl ether/water/methanol (3:3:1) in each Eppendorf tube® (VWR, UK) containing ulcer fluid absorbed into filter discs. Three blank filter disc samples were also prepared in parallel with all of the samples. Blank samples (n=3) containing only the mixture of solvents were also used to permit identification of contaminants introduced by the solvents and tubes. The samples were vortexed for 1 min, then placed in an ultrasound bath for 10 min, followed by centrifugation at 20,000 x g for 30 min at 4 °C. Then the two layers (aqueous and organic) were isolated and this step was repeated one more time in order to increase the recovery of the extraction step. The extracts were dried in a speed vacuum at room temperature and then stored in a -40 °C freezer pending NMR analysis. The organic extracts were allowed to evaporate overnight at room temperature in a fume hood and then stored at -40 °C prior to MS analysis.

All solvents used for the extraction of metabolites from the ulcer fluid and filter disks were obtained from Sigma-Aldrich (Missouri, USA) and were of HPLC Analytical Grade.

**NMR metabolic profiling of the aqueous extracts**

Briefly, ulcer fluid disks aqueous extracts were prepared by the addition of 600 μL of phosphate buffer made up in deuterium oxide containing 1 mM 3-(trimethylsilyl)-[2,2,3,3,2H4] propionic acid sodium salt (TSP) as an external reference and 2 mM sodium azide as a bacteriocide. This was followed by centrifugation (Eppendorf, Centrifuge 5417R, Germany) at 20,000 x g for 10 min at 4 °C. ^1^H NMR spectra were acquired with a Bruker Avance 600 MHz spectrometer operating at 600.13 MHz for 1H at 300 K. It was equipped with a 5 mm broad-band inverse configuration probe. Samples were randomly analysed in automation with a B-ACS 60 sample changer system. For each aqueous extract a standard one-dimensional NMR spectrum was acquired with water peak suppression using a standard pulse sequence (recycle delay (RD)-90o-t1-90o-tm-90o- acquire free induction decay (FID)). The RD was set at 2s, the spin echo delay at 400 µs, the acquisition time per scan was 1.36s and, for each sample, 8 dummy scans were followed by 256 transients. The spectra were collected into 64K data points using a spectral width of 20 ppm.

**RP-UPLC-MS lipid profiling of the organic extracts**

The organic extracts of ulcer fluid and ulcer fluid-disks were reconstituted in a mixture of isopropanol/ acetonitrile/ water (2:1:1, 200 μL), vortexed for 30sec, sonicated for 5min and vortexed for 30sec. This was followed by centrifugation at 20,000 x g for 30 minutes at 4 °C. The supernatant from each sample was transferred into glass inserts in the LC-MS vials. A QC sample was prepared with 30 μL of each sample and was used to assess the analytical reproducibility of the method ^66^. First the column was conditioned by injecting the QC pooled sample, several times, until data showed adequate stability. Afterwards the QC sample was injected every 10 samples to monitor instruments performance. The run was completed by the analysis of extraction and solvent blank samples.

A reversed-phase UPLC-MS method was applied for the untargeted lipid profiling of the organic extracts obtained from ulcer fluid samples. The method has shown excellent retention time repeatability and demonstrated separation of structural isomers. This method is also described in Waters application note “Lipid Separation using UPLC with Charged Surface Hybrid Technology”. The method was performed on an Acquity UPLC system (Waters Corp, USA) coupled to a QTof Premier MS system (Waters MS Technologies, UK). Chromatography was performed using an Acquity UPLC CSH C18 2.1x100mm, 1.7μm, column (Waters Corporation, USA) held at 55 ^o^C. Separation was achieved using gradient elution with 0.1% (v/v) formic acid in acetonitrile/water (60:40) (A) and 0.1% (v/v) in isopropanol/acetonitrile (B) (90:10) at a flow rate of 0.4 mL/min. In both mobile phases ammonium formate (LC-MS grade, Fluka, USA) was diluted to 10mM. Starting conditions were 60% A and 40% B for 2.0 min, changing linearly to 43% B over the next 2 min, and then to 50% B within 0.1 min and to 54% B over the next 10min, when it was changed to 70%B within 0.1 min and finally to 99% B over the next 6 min. Afterward the solvent composition returned to starting conditions over 0.1 min, followed by re- equilibration for 2 min prior to the next injection.

Mass spectrometry was performed using electrospray in both positive and negative ESI ionization modes. The capillary voltage was 1.5 kV in both ESI+ and ESI−, cone voltage was 30 V, desolvation temperature was 600 °C, and source temperature was 120 °C. The cone gas flow rate was 50 L/h, and desolvation gas flow rate was 1,000 L/h. The XEVO G2 Q-TOF was operated in sensitivity mode with a scan time of 0.2 s and interscan delay of 0.015 s. For mass accuracy, a LockSpray interface was used with at 9 ng/mL leucine enkephalin (555.2645 amu) solution (50/50 ACN/H2O with 0.1% v/v formic acid) at 10 μL/min was used as the lock mass. Data were collected in centroid mode with a scan range of 50−2000 m/z, with lockmass scans collected every 30 s and averaged over 4 scans to perform mass correction.

**Serum Metabolic Profiling Analysis**

**NMR metabolic profiling of serum**

For serum analysis, 300 μL of plasma was added to 300 μL of 0.9 % sodium chloride and centrifuged at 20,000 x g for 10 min at 4°C. Aliquots of the plasma solution (550 μL) from each sample were transferred to 5 mm NMR tubes for 1H NMR analysis. The serum samples were analysed using CPMG relaxation-editing sequence with presaturation as described above. Moreover serum samples were also analysed using water suppressed 1D NMR spectrum using the NOESYPRESAT pulse sequence (256 transients). Irradiation of the solvent (water) resonance was applied during presaturation delay (2.0 s) for all spectra and for the water suppressed 1D NMR spectra also during the mixing time (0.1 s). The pulse sequence parameters including the 90° pulse (~ 12 μs), pulse frequency offset (~ 2,800 Hz), receiver gain (~ 90), and pulse powers were optimised for each sample set run. The spectral width was 20 ppm for all spectra. The NMR was processed with an exponential line broadening of 0.3 Hz prior to Fourier transformation, which was collected with approximately 64 k real data points.

**RP-UPLC-MS lipid profiling of serum**

Serum samples are prepared by isopropanol precipitation. Serum, QC and Blank (high purity water) samples were simultaneously prepared by adding chilled isopropanol in a ratio of 3:1; isopropanol: serum, then vortexed and stored cold at -20 ̊C for 1h following centrifugation at 20,000 x g for 30 minutes at 4°C, and then aliquot 150 μl supernatant to glass inserts in MS vials. A QC sample was prepared with 50 μl of each sample and was used to assess the analytical reproducibility of the method. A “blank sample” was also prepared. In this way we will be able to see the possible contamination that comes from the sample preparation procedure.

Chromatographic separation was conducted using an Acquity UPLC system (Waters Ltd, 26 UK) coupled to a XEVO G2 Q-TOF mass spectrometry (Waters Corporation, Milford, U.S.A.) using an ESI interface. The details of the method used for the RP-UPLC-MS lipid profiling of the serum in both positive and negative ESI mode are the same as described above in the section of ulcer fluid metabolic profiling analysis of “RP-UPLC-MS lipid profiling of the organic extracts”.

**HILIC-UPLC-MS profiling of serum**

The sample preparation was carried out by acidic solvent extraction, followed by glycerophospholipid solid-phase extraction (SPE), using Ostro 96-well plates with pressure valves (Ostro plates, Waters). An Ostro plate was fixed on top of a 96-well collection plate in a manifold with a pressure gauge, and was connected to vacuum. Samples (50 μL) were pipetted into the wells, followed by the forceful addition of 1% formic acid in acetonitrile (3:1 solvent/sample) for in-well protein precipitation. The mixture was then quickly mixed to promote solubilization of serum components from the serum protein precipitate, and kept at -20°C for 20 min to promote further protein precipitation. Vacuum (15 in. (∼381 mm) Hg) was then applied to the Ostro plate for 10 min, through a vacuum manifold, causing the valve mat tips in the plate to open; the precipitation solvent containing the nonphospholipid serum components was filtered out and collected in the 96- well collection plate. Then samples were transferred to MS vials with inserts. All solvents were kept at 4 °C prior to their use.

Metabolic profiling was performed on an Acquity UPLC system (Waters Ltd. Elstree, U.K.) coupled to a XEVO G2 Q-TOF mass spectrometer (Waters MS Technologies, Ltd., Manchester, U.K.). Chromatography was performed using an Acquity BEH HILIC (1.7 μm, 2.1 × 100 mm) column (Waters Corporation, Milford, U.S.A.) kept at 40 °C. UPLC separation was conducted using an Acquity UPLC System (Waters Corp, USA). Column temperature was set at 40 °C. Mobile phase A consisted of acetonitrile (ACN)/water (95:5) and mobile phase B ACN/water (50:50). In both solutions ammonium acetate was diluted to 10 mM and formic acid to 0.1%. The elution gradient was set as follows: 99% A (0.0−2.0 min; 0.4 mL/min), 99−45% A (2.0−8.0 min; 0.4 mL/min), 45−1% A (8.0−9.0 min; 0.4 mL/min), 1% A (9.0−9.1 min; 0.4−0.6 mL/min), 1% A (9.1−11.1 min; 0.6 mL/min), 1−99% A (11.1−11.2 min; 0.65 mL/min), 99% A (11.1−11.7 min; 0.65-0.9 mL/min), 99% A (11.7−17.5 min; 0.9 mL/min), 99% A (17.5−17.8 min; 0.9−0.4 mL/min), 99% A (17.8−20.0 min; 0.4 mL/min).

Mass spectrometry was performed using electrospray in both positive and negative ESI ionization modes. The capillary voltage was 1.5 kV (ESI+ and ESI−), cone voltage was 30 V, desolvation temperature was 600 °C, and source temperature was 120 °C. The cone gas flow rate was 50 L/h, and desolvation gas flow rate was 1,000 L/h. The MS was operated in sensitivity mode with a scan time of 0.2 s. For mass accuracy, a LockSpray interface was used with a 9 μg/L leucine enkephalin (555.2645 amu) solution (50/50 ACN/H2O with 0.1% v/v formic acid) at 10 μL/min was used as the lock mass. Data were collected in centroid mode with a scan range of 50−1200 m/z, with lockmass scans collected every 30 s and averaged over 4 scans to perform mass correction.

**Urine Metabolic Profiling Analysis**

**NMR metabolic profiling of urine**

An aliquot of 540 μL for each urine sample was mixed with 60 μL of 0.075M phosphate D_2_O buffer (17% D_2_O, pH 7.4) containing 0.1% TSP. The samples were vortexed and allowed to stand for 10 min prior to centrifugation at 14,000 rpm for 10 min at 4 °C in order to remove insoluble material. Aliquots of the supernatants (550 μL) from each urine sample were pipetted into 5 mm NMR tubes for ^1^H NMR analysis. ^1^H NMR spectra were acquired with a Bruker Avance 600 MHz spectrometer operating at 600.13 MHz for 1H at 300 K. It was equipped with a 5 mm broad-band inverse configuration probe. Samples were randomly analysed in automation with a B-ACS 60 sample changer system. All samples were analysed using a Carr- Purcell-Meiboom-Gill (CPMG) relaxation-editing sequence with presaturation. The sequence attenuates the signal of any remaining large molecules (proteins). It was run with 256 transients, 8 dummy scans, 64k ime domain points, a spectral width of 20 ppm., 2 s relaxation delay, an acquisition time of 1.36s, 80 loops, and a spin echo delay of 400 μs. Irradiation is at the water peak (~ 2800 Hz) during the relaxation delay.

**RP-UPLC-MS profiling of urine**

For RP-UPLC-MS analysis neat urine samples were vortexed and centrifuged without any dilution and aliquoted into 96 well-plates.

Metabolic profiling was performed on an Acquity UPLC system (Waters Ltd. Elstree, U.K.) coupled to a XEVO G2 Q-TOF mass spectrometer (Waters MS Technologies, Ltd., Manchester, U.K.). Chromatography was performed using an Acquity HSS T3 (1.7 μm, 2.1 × 100 mm) column (Waters Corporation, Milford, U.S.A.) Column temperature was set at 40 °C. Mobile phase A consisted of 0.1% formic acid in water and mobile phase B 0.1% formic acid in acetonitrile. The elution gradient was set as follows: 99% A (0.0−1.0 min; 0.5 mL/min), 99−85% A (1.0−3.0 min; 0.5 mL/min), 85−50% A (3.0−6.0 min; 0.5 mL/min), 50-5% A (6.0−9.0 min; 0.5 mL/min), 5% A (9.0−10.0 min; 0.5 mL/min), 5−99% A (10.0−10.1 min; 0.5 mL/min).

Mass spectrometry was performed using electrospray in both positive and negative ESI ionization modes. The capillary voltage was 1.5 kV (ESI+ and ESI−), cone voltage was 30 V, desolvation temperature was 600 °C, and source temperature was 120 °C. The cone gas flow rate was 50 L/h, and desolvation gas flow rate was 1,000 L/h. The MS was operated in sensitivity mode with a scan time of 0.2 s. For mass accuracy, a LockSpray interface was used with a 9 μg/L leucine enkephalin (555.2645 amu) solution (50/50 ACN/H_2_O with 0.1% v/v formic acid) at 10 μL/min was used as the lock mass. Data were collected in centroid mode with a scan range of 50−1200 m/z, with lockmass scans collected every 30 s and averaged over 4 scans to perform mass correction.

**HILIC-UPLC-MS profiling of urine**

For HILIC-UPLC-MS analysis neat urine samples were vortexed and centrifuged without any dilution and aliquoted into 96 well-plates. The HILIC-UPLC-MS method used for the analysis of the urine samples was the same as in the other biofluids.

**NMR Data Extraction.** Data [-1.0 to 10.0 ppm] were imported into MATLAB 7.0 software (MathWorks, Natick, MA), where they were automatically phased, corrected for baseline distortions and referenced to the TSP signal at δ 0.00. The region between δ 4.67- 5.0 containing the residual water resonance was removed from all spectra. Assignment of endogenous ulcer fluid metabolites was made by reference to published literature data and in-house databases.

NMR acquired spectra were imported into Matlab (R2013a, Mathworks, Natick, MA) using in-house routines (MetaSpectra, Dr O. Cloarec) for full resolution introduction of the spectra into Matlab, and a vector running from 0 to 10 ppm, in increments of 0.00025 ppm, was formulated. Using this approach we were able to maintain the actual high resolution of the original spectra and have excellent accuracy in chemical shift referencing. After removal of the area corresponding to residual water (4.40−5.2 ppm), the remaining data were aligned and then probabilistic quotient normalization was applied using in-house Matlab routines.

**MS Data Extraction.** The MS raw data were converted to netCDF format using the DataBridge tool implemented in MassLynxTM software (Waters Corporation, Milford, USA). The data were processed using the freely available package in R programming software ^67,68^ and an output table was obtained comprising pairs of m/z_RT and intensity values of the detected metabolite features in each sample. The dataset then was normalized with the media fold change method in R with an in-house script ^69^.

**Statistical Analysis.** Prior to statistical analysis datasets were normalised to total area. The purpose of normalisation is to remove sources of systematic variation between samples that are irrelevant in regard to the study objectives, such as sample dilution or technical variation (e.g. sample preparation/analytical platform variation). This should allow biological variation of interest to be observed more clearly.

Multivariate data analysis was performed using the SIMCA package (v.13.0.2, Umetrics, Umeå, Sweden). Principal component analysis (PCA) was used to visualize data and assess the reproducibility of the methodologies. Also, orthogonal projection to latent structures discriminant analysis (OPLS-DA) and orthogonal projection to latent structures (OPLS) modelling were used to examine data in a multivariate setting. Prior to model fitting, features were subjected to Pareto scaling. Validity of the models was inspected through cross-validation test and permutation test. S-plot and S-line were plotted to identify discriminatory metabolites for MS and NMR dataset, respectively.

**2. Supplemental Figures**

**Principal Component Analysis (PCA) Plots**

Supplemental Figure 1 - PCA plot for serum samples, HILIC profiling positive mode (HILIC-MS ESI+).

HILIC – hydrophilic interaction liquid chromatography; MS – mass spectrometry; ESI – electrospray ionisation (+/- positive and negative mode); R^2^Y – variation in the matrices explained by the model; Q^2^Y – predictive ability of the model; QC – quality control sample.


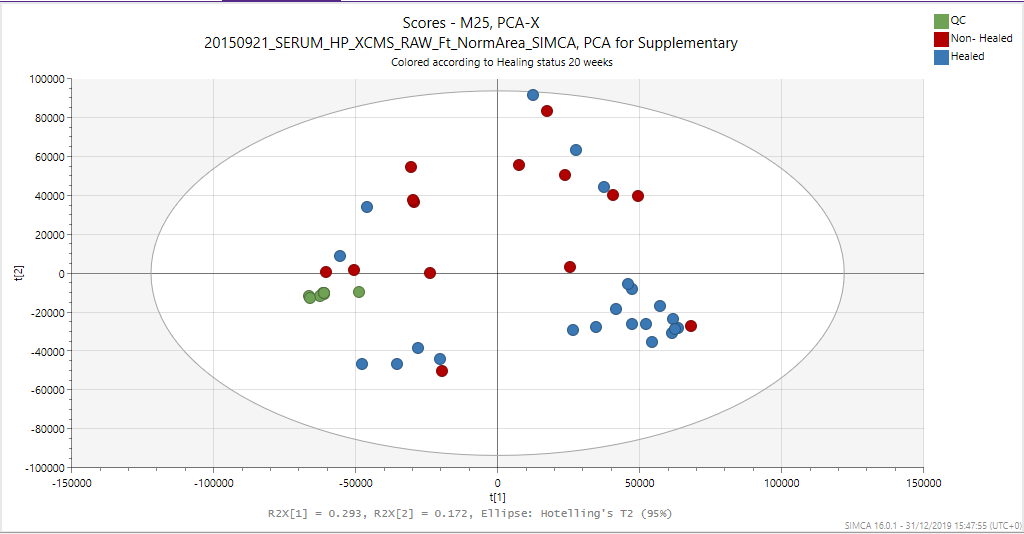


Supplemental Figure 2 - PCA plot for serum samples, HILIC profiling negative mode (HILIC-MS ESI-).

HILIC – hydrophilic interaction liquid chromatography; MS – mass spectrometry; ESI – electrospray ionisation (+/- positive and negative mode); R^2^Y – variation in the matrices explained by the model; Q^2^Y – predictive ability of the model; QC – quality control sample.


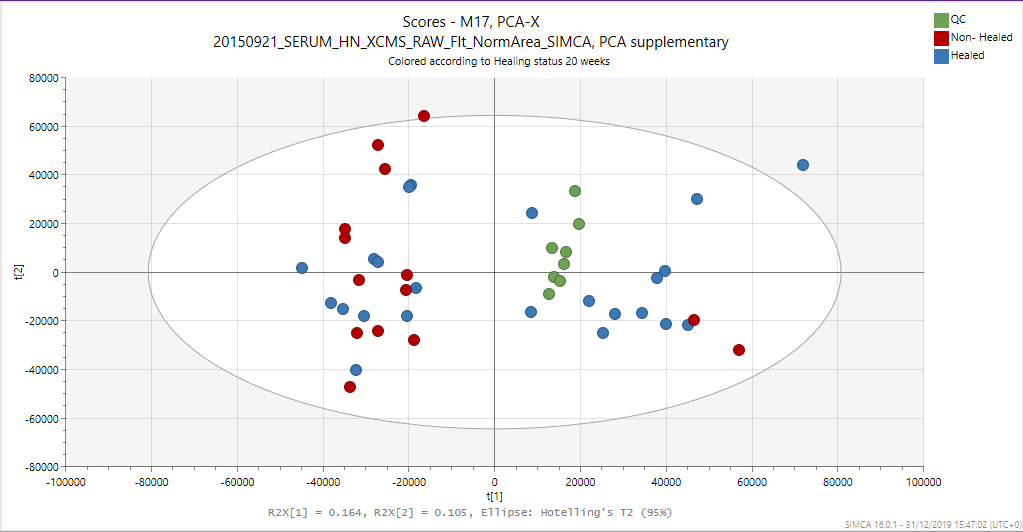


Supplemental Figure 3 - PCA plot for serum samples, reversed phase negative mode (RPLC-MS ESI -).

RPLC – MS – reversed phase liquid chromatography; MS – mass spectrometry; ESI – electrospray ionisation (+/- positive and negative mode); R^2^Y – variation in the matrices explained by the model; Q^2^Y – predictive ability of the model; QC – quality control sample.


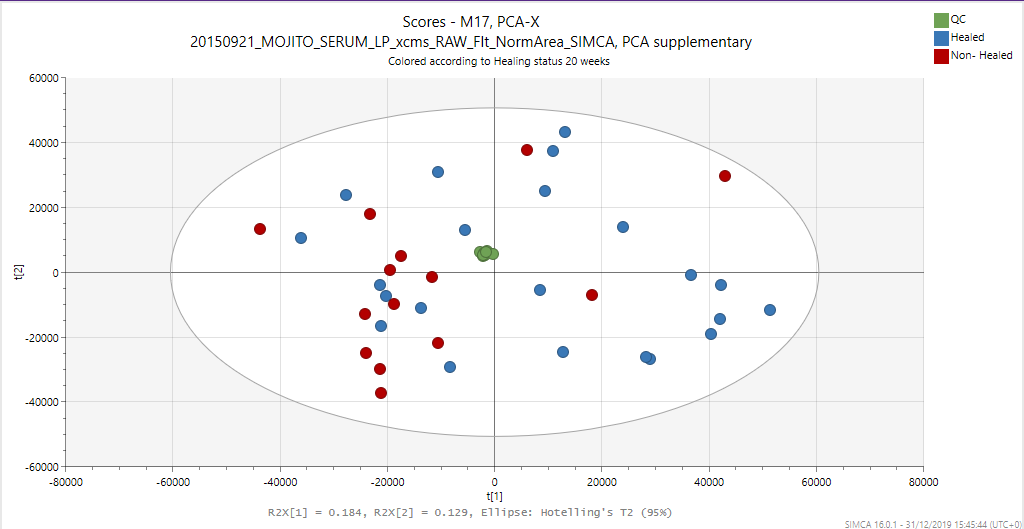


Supplemental Figure 4 - PCA plot for urine samples, reversed phase profiling positive mode (RPLC-MS ESI+).

RPLC – MS – reversed phase liquid chromatography; MS – mass spectrometry; ESI – electrospray ionisation (+/- positive and negative mode); R^2^Y – variation in the matrices explained by the model; Q^2^Y – predictive ability of the model; QC – quality control sample.


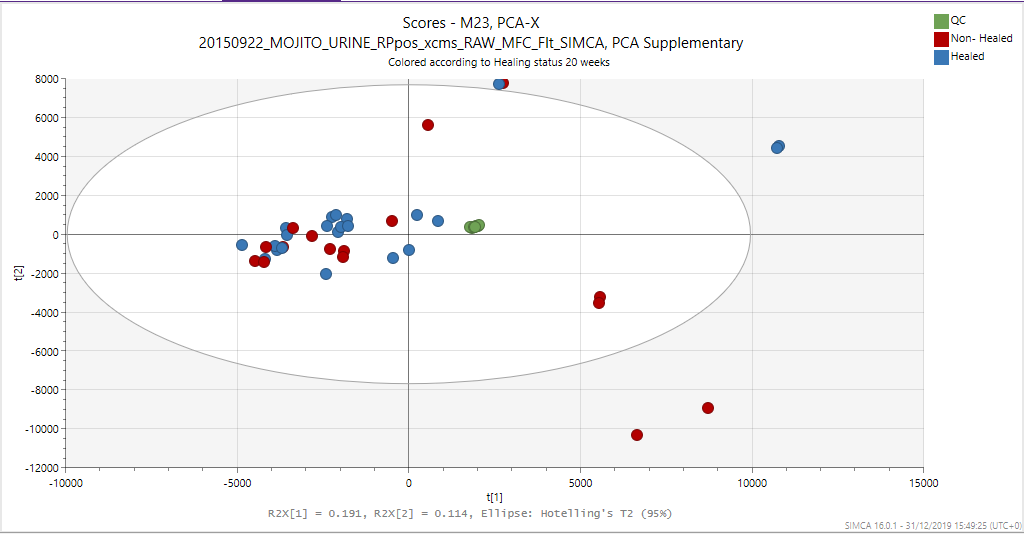


Supplemental Figure 5 - PCA plot for urine samples, reversed phase profiling negative mode (RP neg).

RPLC – MS – reversed phase liquid chromatography; MS – mass spectrometry; ESI – electrospray ionisation (+/- positive and negative mode); R^2^Y – variation in the matrices explained by the model; Q^2^Y – predictive ability of the model; QC – quality control sample.


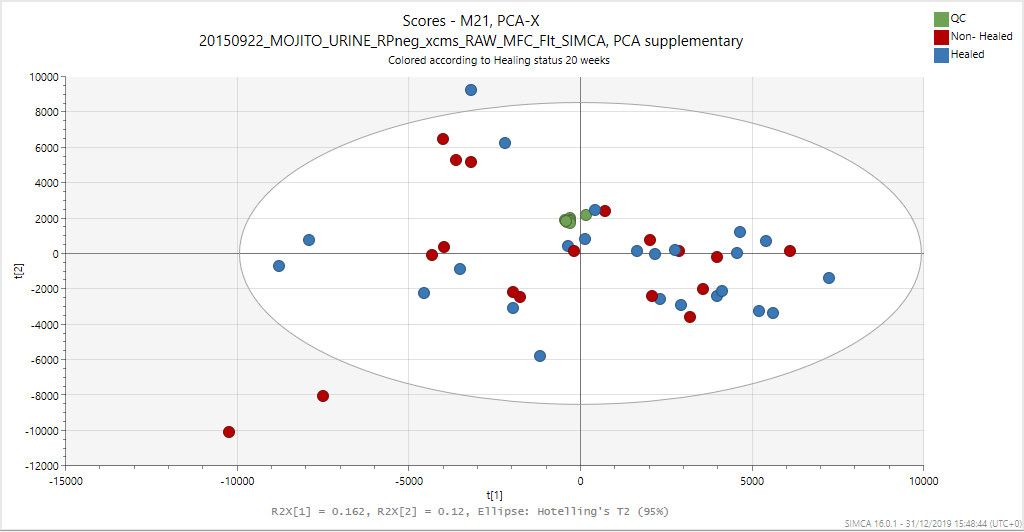


Supplemental Figure 6 – PCA plot for ulcer fluid samples, reversed phase negative mode (RPLC-MS ESI-).

RPLC – MS – reversed phase liquid chromatography; MS – mass spectrometry; ESI – electrospray ionisation (+/- positive and negative mode); R^2^Y – variation in the matrices explained by the model; Q^2^Y – predictive ability of the model; QC – quality control sample.


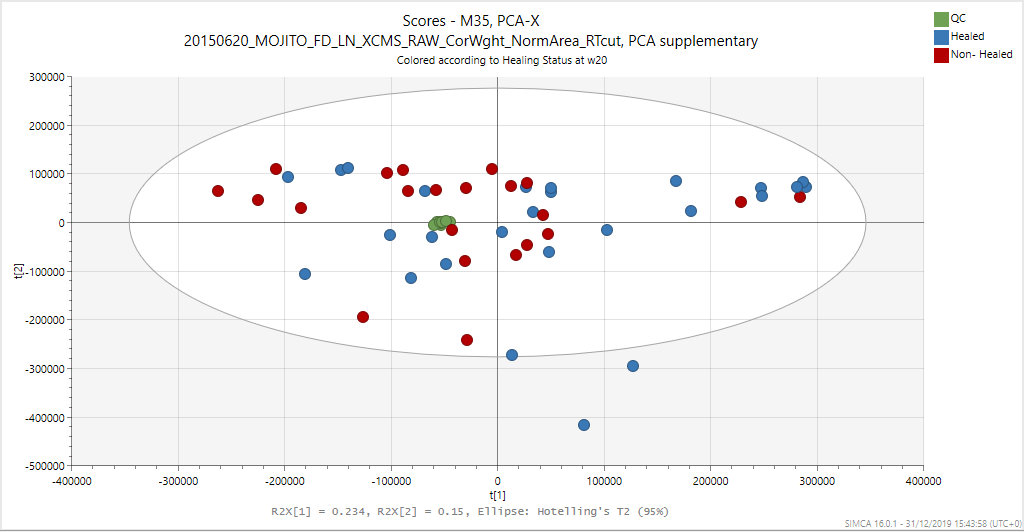


**Supplemental Figure Legends**

**Principal Component Analysis (PCA) Plots**

Supplemental Figure 1 - PCA plot for serum samples, HILIC profiling positive mode (HILIC-MS ESI+).

HILIC – hydrophilic interaction liquid chromatography; MS – mass spectrometry; ESI – electrospray ionisation (+/- positive and negative mode); R^2^Y – variation in the matrices explained by the model; Q^2^Y – predictive ability of the model; QC – quality control sample.

Supplemental Figure 2 - PCA plot for serum samples, HILIC profiling negative mode (HILIC-MS ESI-).

HILIC – hydrophilic interaction liquid chromatography; MS – mass spectrometry; ESI – electrospray ionisation (+/- positive and negative mode); R^2^Y – variation in the matrices explained by the model; Q^2^Y – predictive ability of the model; QC – quality control sample.

Supplemental Figure 3 - PCA plot for serum samples, reversed phase negative mode (RPLC-MS ESI -).

RPLC – MS – reversed phase liquid chromatography; MS – mass spectrometry; ESI – electrospray ionisation (+/- positive and negative mode); R^2^Y – variation in the matrices explained by the model; Q^2^Y – predictive ability of the model; QC – quality control sample.

Supplemental Figure 4 - PCA plot for urine samples, reversed phase profiling positive mode (RPLC-MS ESI+).

RPLC – MS – reversed phase liquid chromatography; MS – mass spectrometry; ESI – electrospray ionisation (+/- positive and negative mode); R^2^Y – variation in the matrices explained by the model; Q^2^Y – predictive ability of the model; QC – quality control sample.

Supplemental Figure 5 - PCA plot for urine samples, reversed phase profiling negative mode (RP neg).

RPLC – MS – reversed phase liquid chromatography; MS – mass spectrometry; ESI – electrospray ionisation (+/- positive and negative mode); R^2^Y – variation in the matrices explained by the model; Q^2^Y – predictive ability of the model; QC – quality control sample.

Supplemental Figure 6 – PCA plot for ulcer fluid samples, reversed phase negative mode (RPLC-MS ESI-).

RPLC – MS – reversed phase liquid chromatography; MS – mass spectrometry; ESI – electrospray ionisation (+/- positive and negative mode); R^2^Y – variation in the matrices explained by the model; Q^2^Y – predictive ability of the model; QC – quality control sample.
